# Decline in Serum Lysophosphatidylcholine Species in Patients with Severe Inflammatory Bowel Disease

**DOI:** 10.3390/jcm14155485

**Published:** 2025-08-04

**Authors:** Hauke Christian Tews, Tanja Elger, Muriel Huss, Johanna Loibl, Arne Kandulski, Martina Müller, Marcus Höring, Gerhard Liebisch, Christa Buechler

**Affiliations:** 1 Department of Internal Medicine I, Gastroenterology, Hepatology, Endocrinology, Rheumatology, and Infectious Diseases, University Hospital Regensburg, 93053 Regensburg, Germany; hauke.tews@klinik.uni-regensburg.de (H.C.T.); muriel.huss@klinik.uni-regensburg.de (M.H.); johanna.loibl@klinik.uni-regensburg.de (J.L.); arne.kandulski@klinik.uni-regensburg.de (A.K.); martina.mueller-schilling@klinik.uni-regensburg.de (M.M.); 2 Institute of Clinical Chemistry and Laboratory Medicine, University Hospital Regensburg, 93053 Regensburg, Germany; marcus.hoering@klinik.uni-regensburg.de (M.H.); gerhard.liebisch@klinik.uni-regensburg.de (G.L.)

**Keywords:** Crohn’s disease, ulcerative colitis, lysophosphatidylcholine, corticosteroid, biomarker

## Abstract

**Background/Objectives**: Lysophosphatidylcholine (LPC) is composed of various lipid species, some of which exert pro-inflammatory and others anti-inflammatory activities. However, most of the LPC species analyzed to date are reduced in the serum of patients with inflammatory bowel disease (IBD) compared to healthy controls. To our knowledge, the correlation between serum LPC species levels and measures of inflammation, as well as their potential as markers for monitoring IBD activity, has not yet been investigated. **Methods**: Thirteen LPC species, varying in acyl chain length and number of double bonds, were measured in the serum of 16 controls and the serum of 57 patients with IBD. Associations with C-reactive protein (CRP) and fecal calprotectin levels as markers of IBD severity were assessed. **Results**: Serum levels of LPC species did not differ between the healthy controls and the entire patient cohort. In patients with IBD, serum levels of LPC 16:1, 18:0, 18:3, 20:3, and 20:5, as well as total LPC concentrations, showed inverse correlations with both CRP and fecal calprotectin levels, indicating an association with inflammatory activity. Nine LPC species were significantly reduced in patients with high fecal calprotectin compared to those with low values. LPC species with 22 carbon atoms and 4 to 6 double bonds were not related to disease activity. Stool consistency and gastrointestinal symptoms did not influence serum LPC profiles. Corticosteroid treatment was associated with lower serum LPC 20:3 and 22:5 levels, while mesalazine, anti-TNF, and anti-IL-12/23 therapies had no significant impact on LPC concentrations. There was a strong positive correlation between LPC species containing 15 to 18 carbon atoms and serum cholesterol, triglycerides, and phosphatidylcholine levels. However, there was no correlation with markers of liver disease. **Conclusions**: Shorter-chain LPC species are reduced in patients with active IBD and reflect underlying hypolipidemia. While these lipid alterations provide insight into IBD-associated metabolic changes, they appear unsuitable as diagnostic or disease monitoring biomarkers.

## 1. Introduction

Chronic inflammation strongly affects the metabolism of the major lipoproteins, low-density lipoprotein (LDL) and high-density lipoprotein (HDL) [1]. These lipoprotein particles are composed of different lipid classes such as cholesterol, sphingolipids, glycerolipids, and glycerophospholipids [2]. Comprehensive analysis of the serum lipid composition is currently being used to identify biomarkers for disease diagnosis, prognosis, and monitoring in inflammatory diseases [3,4,5].

Inflammatory bowel disease (IBD) is a group of chronic inflammatory conditions that can affect the whole intestinal tract but also associates with extraintestinal manifestations such as the eyes, joints, liver, and kidneys [6]. Lipidomics in IBD has identified biomarkers for IBD diagnosis, differentiation of Crohn’s disease (CD) and ulcerative colitis (UC), and monitoring of disease activity [7,8,9,10,11], but these are not currently used in clinical practice. An important aspect of lipidomic analysis is also to elucidate the pathophysiology of IBD, which is still poorly understood [12,13,14,15]. Such approaches will identify the pathways that contribute to disease progression, which may become targets for treatment.

Lysophosphatidylcholines (LPCs) have long been regarded as inflammatory lipids that also exert cytotoxic effects [16,17]. LPC species vary in both acyl chain length and the number of double bonds, which influences their biological activity [2,18]. Indeed, it has been shown that the pro-inflammatory effects of LPC are mainly achieved by saturated LPC species, such as LPC 16:0, whereas the polyunsaturated LPC species, including LPC 20:4 and LPC 22:6, have anti-inflammatory functions [17]. However, saturated and unsaturated LPC species were reduced in the plasma of patients with sepsis compared to controls [19]. Therefore, analysis of LPC species is recommended to gain insight into their associations with inflammatory processes.

LPC in the blood is associated with HDL and also binds to albumin [2]. The circulating LPC species levels of IBD patients and healthy controls were compared to identify diagnostic biomarkers. A patient cohort consisting of CD and UC cases with different disease activities had lower plasma LPC 18:0, 18:1, and 18:2 compared to healthy controls [20]. Similarly, plasma LPC 20:4, 22:1, and 24:1 were lower in IBD patients than in healthy controls [21]. Plasma LPC 18:2 was also found to be reduced in patients with active IBD compared to healthy controls, whereas LPC 20:4 levels of the patients were increased [22]. In newly diagnosed children with IBD, serum LPC levels were lower than those of the healthy controls, and LPC 18:3, 20:5, 22:5, and 22:6 were reduced in CD and UC compared to controls. The decline in LPC 17:1, 20:2, 20:3, 20:4, and 22:4 was only observed in UC, but not in patients with CD, compared to the controls. However, LPC species levels did not differ significantly between CD and UC patients [23]. Current evidence suggests that LPC species, including saturated and polyunsaturated lipids, are mostly reduced in the circulation of IBD patients compared to healthy controls.

Lecithin–cholesterol acyltransferase hydrolysis of phosphatidylcholine produces LPC [24] (Figure S1). LPC is also derived from phosphatidylcholine by secretory phospholipase A2 cleavage [17] (Figure S1). Secretory phospholipase A2 contributes to the pathophysiology of IBD, and inhibition of this enzyme improved the severity of colitis in an experimental model [25]. Sulfasalazine, an anti-inflammatory drug used in IBD therapy, inhibits the release of secretory phospholipase A2 [26], but it is unknown whether reduced production of LPC is also an effect of this drug. Glucocorticoids, used to treat flares and induce remission in IBD [27], also inhibit the induction of secretory phospholipase A2 by tumor necrosis factor [28]. Mesalazine (5-aminosalicylic acid), which is also used in IBD therapy, has no effect on phospholipase A2 [29]. Circulating levels of secretory phospholipase A2 are also reduced by anti-TNF antibody infusion in patients with active CD [30]. Given the increased activity of secretory phospholipase A2 in patients with IBD [30], the pathways leading to low blood levels of LPC [7,31] are unclear.

Patients with active IBD commonly exhibit elevated serum C-reactive protein (CRP), the most widely applied systemic marker of inflammation [32,33,34]. Fecal calprotectin, a non-invasive marker of mucosal inflammation, is also widely used to monitor IBD activity [35,36]. However, both CRP and fecal calprotectin increase in various inflammatory conditions and lack disease specificity [32,35,36]. Despite this limitation, they remain valuable tools for assessing disease activity in IBD [32,35,36]. While LPC species have been implicated in inflammatory processes, their relationship with established IBD activity markers has not yet been systematically explored.

The primary objective of this study was to investigate the associations between serum LPC species levels and markers of systemic (CRP) and mucosal (fecal calprotectin) inflammation in patients with IBD. Additionally, we evaluated the potential impact of current IBD therapies on circulating LPC profiles. Given the metabolic links between LPC and other lipid classes, correlations with serum cholesterol, triglycerides, phosphatidylcholine, and clinical markers of liver function were also examined.

## 2. Materials and Methods

### 2.1. Patients

Adult patients with IBD, confirmed by histology, endoscopy, and clinical assessment [37], were randomly included in the study. From 6 December 2021 to 31 January 2023, patients were asked for their consent to provide serum for the study at the Department of Internal Medicine I, University Hospital Regensburg. Patients with coagulopathy, primary sclerosing cholangitis, and pregnant women were excluded from this study cohort. Endoscopic scores were not consistently available at the time of blood sampling and were therefore not included in the analyses.

### 2.2. Measurement of Serum LPC Species

Serum was mixed with 25 µL of internal standard solution containing LPC 13:0 (0.83 nmol) and LPC 19:0 (0.7 nmol) and vacuum-dried. LPC 16:0, 18:0, and LPC 18:1, which are 1-acyl-2-hydroxy-sn-glycero-3-phosphocholines (Avanti Polar Lipids, Alabaster, AL, USA), were used to generate matrix-based calibration lines. LPC species levels were determined by direct electrospray ionization tandem mass spectrometry (ESI-MS/MS) [38]. A volume of 10 µL serum was extracted according to the protocol of Bligh and Dyer [39]. The chloroform phase was vacuum-dried, solubilized in 7.5 mM ammonium acetate in methanol–chloroform (3:1 by volume). The triple quadrupole mass spectrometer (Quattro LC; Micromass; Manchester, UK) was operated in positive selected reaction monitoring using a product ion of *m*/*z* 184. The mass spectrometer was used with the following settings: ionization voltage: 3500 V; source temperature: 300 °C; cone voltage of 41 V; collision energy of 24 V; and unit resolution for both Q1 and Q3. Details of the MS settings, as well as an example mass spectrum showing the relevant LPC species in serum, have been described previously [38].

The data were analyzed using the MassLynx software V4.2 (Waters GmbH, Eschborn, Germany) and also using the NeoLynx tool to obtain averages of the scans at half peak height of the total ion count. The results were exported to MS Excel spreadsheets and were further processed using self-programmed macros to sort the results, correct for isotopic overlap (Type II) [40], and calculate ratios to the internal standards. Calibration curves and quantitative values were also obtained. The methods used for the analyses of cholesterol [41], phosphatidylcholine [42], and triglycerides [43] have been described before.

### 2.3. Statistical Analysis

Table 1 describes the statistical tests used and how the data are presented in the figures and tables.

## 3. Results

### 3.1. Study Cohort

The study included 26 female and 31 male patients with IBD; 40 had CD, and 17 had UC (Table S1). Eleven women and five men served as healthy controls (*p* = 0.087 for sex distribution of cases and controls). The age of the controls was 52 (24–78) years, which was similar to that of the patients (*p* = 0.120). The controls were all healthy and had a normal body mass index (BMI). Laboratory measurements of the controls were not recorded. Details of the cases are summarized in Table 2.

LPC species levels were similar in the male and female IBD patients. LPC 18:0 was the only LPC species that significantly correlated with age in the entire patient cohort (r = 0.452, *p* = 0.005; Table S2). None of the LPC species were associated with BMI (Table S2).

None of the LPC species was significantly reduced in the patients compared to the controls (Table 3). LPC species levels of the patients with CD and UC were comparable (Table S3). It should be noted that CRP and fecal calprotectin of the patients with CD and UC did not differ (Table S1).

### 3.2. Correlation of LPC Species with CRP and Fecal Calprotectin

**Figure 1:**
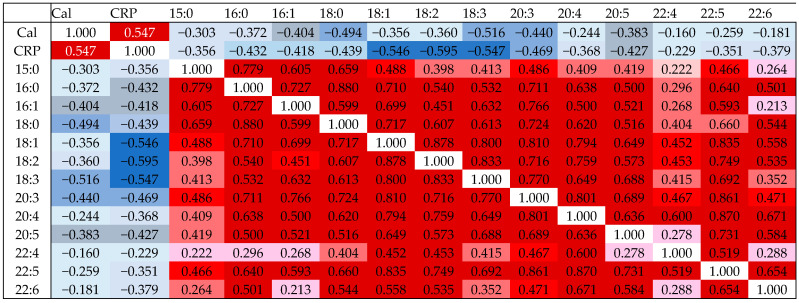
Correlation of LPC species with each other, C-reactive protein (CRP), and fecal calprotectin (Cal). Spearman’s correlation coefficients are shown in the table. The blue color signifies a negative correlation, with the color intensity reflecting the strength of the correlation. The red color signifies a positive correlation with the color intensity reflecting the strength of the correlation.

The Spearman correlation analysis showed that in patients with IBD, all LPCs except LPC 15:0, 20:4, 22:4, 22:5, and 22:6 were negatively correlated with CRP (Table S4 and Figure 1). Negative correlations between fecal calprotectin and LPC 16:1, 18:0, 18:3, 20:3, and 20:5 were also significant (Table S4 and Figure 1). The total serum LPC levels were negatively correlated with CRP and fecal calprotectin (Table S4).

LPC species levels positively correlated with each other (Figure 1). LPC 22:4 did not correlate with LPC 15:0, 16:0, 16:1, 20:5, and 22:6, and LPC 22:6 did not correlate with LPC 15:0, 16:1, and 22:4 (Figure 1).

When the correlations with CRP and fecal calprotectin were analyzed in the patients with CD, LPC 18:1 and LPC 18:2 were found to negatively correlate with CRP, while LPC 18:3 was found to negatively correlate with calprotectin (Table S5). In the patients with UC, LPC 15:0, 16:0, 18:0, 18:1, 18:2, 18:3, 20:3, 20:4, 20:5, and 22:6 negatively correlated with CRP, while LPC 18:0 correlated with calprotectin (Table S5).

**Figure 2:**
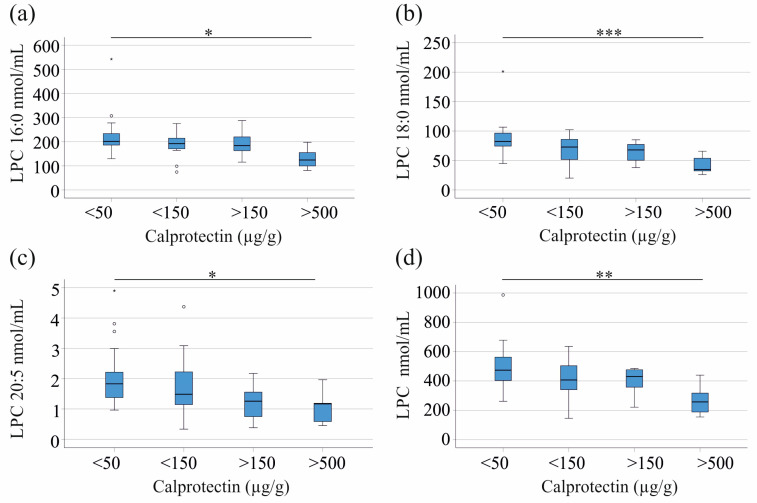
Serum LPC levels of patients with increasing fecal calprotectin as a marker of disease activity: (**a**) LPC 16:0; (**b**) LPC 18:0; (**c**) LPC 20:5; and (**d**) total serum LPC levels of IBD patients stratified for fecal calprotectin levels. * *p* < 0.05, ** *p* < 0.01, and *** *p* < 0.001.

In the entire cohort, LPC 15:0 (*p* = 0.010), 16:0 (*p* = 0.011), 16:1 (*p* = 0.010), 18:0 (*p* < 0.001), 18:1 (*p* = 0.021), 18:2 (*p* = 0.012), 18:3 (*p* = 0.001), 20:3 (*p* = 0.004), 20:5 (*p* = 0.039), and total LPC levels (*p* = 0.004) were significantly higher in the 27 patients with calprotectin levels below 50 µg/g compared to the 7 patients with fecal calprotectin levels above 500 µg/g (Figure 2a–d and Figure S2a–f). The 16 patients with calprotectin levels between 50 and 150 µg/g and the 7 patients with levels between 150 and 500 µg/g did not differ from the patients with low and high calprotectin levels (Figure 2a–d and Figure S2a–f). This analysis was not conducted separately for the patients with CD and UC because there were too few patients for meaningful statistical tests to be conducted.

**Figure 3:**
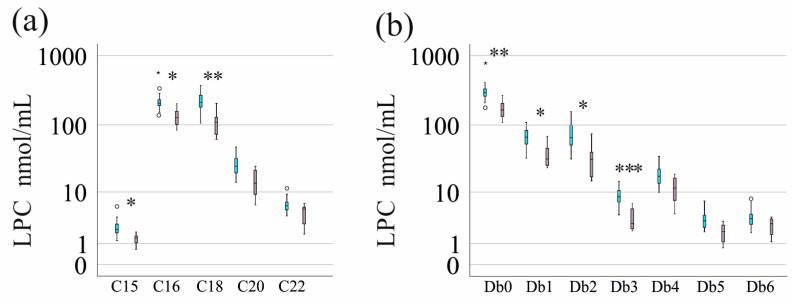
Serum LPC levels in patients with low (turquoise) and high (pink) fecal calprotectin as a marker of disease activity: (**a**) LPC species with an identical number of carbon atoms (C15, C16, C18, C20, C22) are shown; (**b**) LPC species with an identical number of double bonds (Db0–Db6) are shown. * *p* < 0.05, ** *p* < 0.01, and *** *p* < 0.001.

LPC species with 15, 16, and 18 carbon atoms but not those with 20 or 22 carbon atoms were significantly reduced in the serum of patients with severe IBD (Figure 3). Accordingly, LPCs with four or five double bonds were not related to fecal calprotectin levels, whereas LPCs with fewer double bonds in patients with high fecal calprotectin were lower (Figure 3).

### 3.3. Correlation of LPC Species with Measures of Liver Function

Serum LPC levels are reduced in patients with liver cirrhosis and show an association with liver function [45]. Aspartate aminotransferase, alanine aminotransferase, gamma-glutamyl transferase, bilirubin, and alkaline phosphatase did not correlate with any of the LPC species (*p* > 0.05 for all), excluding a close association with liver function.

### 3.4. LPC Species Levels in Relation to Current Treatment

**Figure 4:**
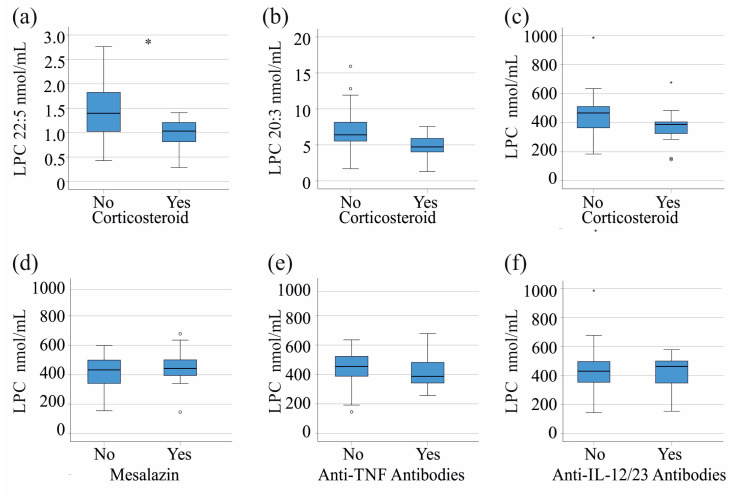
Serum LPC levels of patients with different treatments: (**a**) LPC 22:5, (**b**) LPC 20:3, and (**c**) total LPC levels of patients with (Yes) and without (No) corticosteroid therapy; total serum LPC levels of IBD patients treated with (**d**) mesalazine, (**e**) anti-TNF antibodies, or (**f**) anti-IL-12/23 antibodies in comparison to patients on other medications. * *p* < 0.05.

The 14 patients currently treated with corticosteroids had lower levels of LPC 22:5 (*p* = 0.026, Figure 4a) and a trend toward lower levels of LPC 20:3 (*p* = 0.052, Figure 4b). All other LPC species and total serum LPC levels (Figure 4c) did not differ between the groups. Age, BMI, CRP, calprotectin, serum cholesterol, triglyceride, and phosphatidylcholine levels did not change in the patients with and without corticosteroid treatment (*p* > 0.05 for all). Sex distribution and prevalence of CD and UC were comparable between the groups (*p* > 0.05). The 14 patients on mesalazine (Figure 4d), the 15 patients on anti-tumor necrosis factor therapy (Figure 4e), and the 13 patients on anti-IL-12/23 therapy (Figure 4f) had serum LPC levels, and CRP and fecal calprotectin levels similar to those of patients on other medications (*p* > 0.05 for all). It should be noted that almost all of our patients were treated with a combination of drugs, which may limit the validity of this analysis. It was not possible to analyze the association of LPC species levels with defined drug combinations because the cohort was too small.

### 3.5. LPC Species Levels in Relation to Serum Cholesterol, Triglyceride, and Phosphatidylcholine Levels

Serum LPC is bound to albumin but is also associated with lipoprotein particles [2]. Serum cholesterol levels of patients with IBD decrease in parallel with disease activity [46] and were negatively correlated with CRP and fecal calprotectin (Table 4). The total triglyceride levels were not reduced in severe IBD (*p* > 0.05) and did not correlate with CRP and fecal calprotectin (Table 4).

All LPC species but LPC 20:4, 20:5, 22:4, and 22:5 were positively correlated with cholesterol. LPC 15:0, 16:0, 16:1, 18:0, 20:3, and 22:5 positively correlated with triglycerides (Table 4).

In CD, LPC 16:0, and in UC, all LPC species except LPC 18:1, 20:5, 22:4, 22:5, and 22:6 were positively correlated with cholesterol (Table S6). LPC 16:0, 16:1, and 18:0 were positively correlated with triglycerides in CD. LPC species were not associated with triglycerides in patients with UC (Table S6).

**Figure 5:**
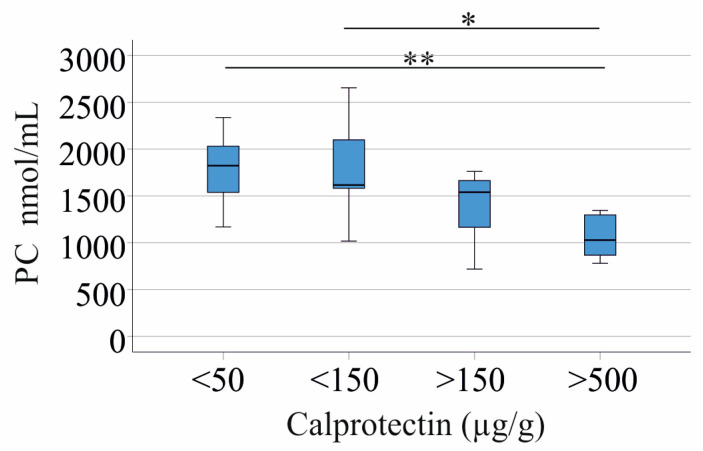
Serum phosphatidylcholine (PC) levels of patients with increasing fecal calprotectin as a marker of disease activity. PC levels of IBD patients stratified for fecal calprotectin levels. * *p* < 0.05, ** *p* < 0.01.

LPC is derived from phosphatidylcholine (PC) [47], and the total PC levels of patients with IBD and controls were similar (*p* > 0.05). Notably, the total PC levels also declined with higher inflammation (Figure 5) and were negatively correlated with CRP and calprotectin (Table 4). The LPC/PC ratio was not related to the fecal calprotectin levels (*p* > 0.05).

In CD, LPC 18:0 positively correlated with phosphatidylcholine, and all LPC species except LPC 20:4, 22:4, 22:5, and 22:6 positively correlated with phosphatidylcholine in UC (Table S6). The association of phosphatidylcholine with CRP and calprotectin was significant in UC but not in CD (Table S6).

In the entire cohort, LPC species did not correlate with CRP or fecal calprotectin when adjusted for total cholesterol levels or PC levels (*p* > 0.05 for all). When corrected for triglyceride levels, LPC 18:0 (r = −0.404, *p* = 0.045), LPC 18:1 (r = −0.400, *p* = 0.039), LPC 18:2 (r = −0.442, *p* = 0.012), LPC 18:3 (r = −0.426, *p* = 0.019), and LPC 20:3 (r = −0.406, *p* = 0.033) still correlated with fecal calprotectin. LPC 18:1 (r = −0.425, *p* = 0.020), LPC 18:2 (r = −0.458, *p* = 0.007), and LPC 18:3 (r = −0.428, *p* = 0.018) correlated with CRP.

### 3.6. LPC Species Levels in Relation to Stool Consistency and Gastrointestinal Symptoms

**Figure 6:**
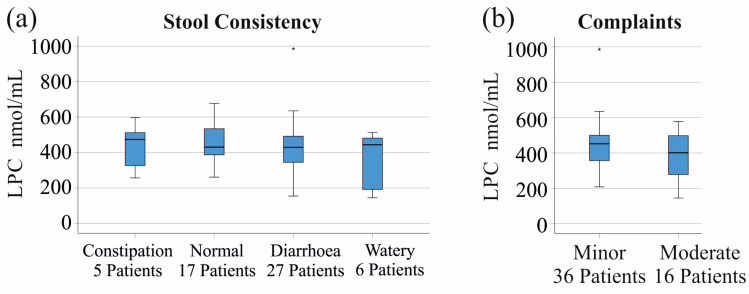
Serum LPC levels of patients in relation to stool consistency and complaints: (**a**) LPC levels of patients with different stool consistencies; (**b**) LPC levels of patients with minor and moderate gastrointestinal complaints.

Stools were scored by 55 patients using the Bristol stool chart. The total LPC (Figure 6a) and LPC species levels were not associated with stool consistency (*p* > 0.05 for all).

Patients with minor and moderate gastrointestinal symptoms had similar levels of all the LPC species analyzed, and accordingly, there was no difference in the total LPC levels (Figure 6b) between these groups. There were two patients without complaints, and two patients with severe symptoms, whose data were not included in the analyses and are not shown in Figure 6b (data of one patient were not recorded). This analysis was not conducted separately for patients with CD and UC because there were too few patients for meaningful statistical tests.

## 4. Discussion

In this study, we demonstrate that mostly LPC species with 15, 16, or 18 carbon atoms were reduced in patients with severe IBD and elevated fecal calprotectin, reflecting active intestinal inflammation. In contrast, patients with mild disease exhibited LPC profiles comparable to healthy controls. These findings provide novel insight into lipid metabolism alterations in active IBD. However, due to the overlap between patients with mild disease and healthy controls, serum LPC species do not appear suitable as stand-alone biomarkers for IBD diagnosis or routine disease monitoring.

A previous study reported reduced serum levels of LPC 18:0, 18:1, and 18:2 in patients with IBD compared to healthy controls [20]. In that cohort, 68 patients presented with moderate or severe disease activity, while 60 were in remission [20]. In contrast, in our cohort, only 14 of the 57 patients exhibited moderate or severe disease, while the majority had mild or inactive disease. Consistent with this difference in disease activity, we did not observe significant reductions in LPC species levels of patients with IBD compared to healthy controls. Our findings suggest that LPC levels remain largely preserved in patients with mild disease activity and that the limited number of patients with active IBD in our cohort likely explains the absence of group differences. Similarly, the study by Tefas et al., which demonstrated a reduction in LPC 18:2 and an increase in LPC 20:4 in IBD compared to healthy controls, did not report on the distribution of disease severity within their study population [22]. In our study, LPC 15:0, 16:0, 16:1, 18:0, 18:1, 18:2, 18:3, 20:3, and 20:5 were found to decrease in patients with very high fecal calprotectin levels (<500 µg/g). Previous studies reported on a decrease in circulating LPC species in IBD patients with severe disease [20,48], which is consistent with our observations.

Notably, LPC species with acyl chain lengths below 22 carbon atoms displayed inverse correlations with inflammatory markers and positive associations with serum cholesterol and phosphatidylcholine levels. In contrast, species such as LPC 20:4, 22:4, 22:5, and 22:6 remained largely unaffected in patients with severe IBD and did not show significant correlations with serum cholesterol, phosphatidylcholine, CRP, or fecal calprotectin. Consistent with previous observations that serum cholesterol levels decrease in active IBD [46] our partial correlation analyses controlling for serum cholesterol revealed that the associations between LPC species and fecal calprotectin lost statistical significance. However, when controlling for triglyceride levels, the correlations between fecal calprotectin and LPC 18:0, 18:1, 18:2, 18:3, and 20:3 remained significant, supporting a partial triglyceride-independent association of specific LPC species with intestinal inflammation.

Notably, associations between LPC species and CRP and fecal calprotectin, as well as between LPC species and cholesterol and phosphatidylcholine levels, were mostly observed in patients with UC compared to patients with CD. However, the UC cohort was small, so this preliminary finding must be confirmed in larger cohorts.

Serum LPC is mostly bound to albumin and HDL [2], and a decrease in HDL levels in severe IBD has been reported in several studies [7]. HDL is a relatively triglyceride-poor particle [49], consistent with normal triglyceride levels in patients with active IBD. About 60% of the serum phosphatidylcholine is found in HDL [2], and when adjusted for PC, serum LPC species were not correlated with inflammation in patients with IBD. These data suggest that low HDL contributes to reduced levels of LPC species in IBD. Preliminary data suggest that HDL is enriched in LPC 16:0 and LPC 18:2 compared to LDL, but this needs further study [2].

Previous studies mostly did not determine phosphatidylcholine in parallel with LPC levels of patients with IBD [21,22]. Our current study showed that serum PC is also reduced in patients with severe IBD (Figure S1). Because the LPC/PC ratio of patients with mild and severe inflammation was similar, processing of PC to LPC in patients with severe IBD is normal.

Hypoalbuminemia in patients with severe IBD may result from increased gastrointestinal loss, which may also be related to lower serum LPC levels [50]. Diarrhea was not associated with altered LPC species levels, but whether albumin levels are low in the patients was not assessed in the current study.

Low albumin is also a marker of impaired hepatocyte synthesis [50]. Serum LPC species levels did not correlate with measures of liver disease, suggesting that liver function, which was quite normal in our cohort, is not a confounding factor. It should be mentioned that patients with liver cirrhosis have low systemic levels of almost all LPC species [51], showing that LPCs released by the liver significantly contribute to its circulating levels. Overall, the low level of HDL and LDL particles is likely to contribute to the reduced level of LPC in IBD [10,33].

Inflammatory diseases are linked with reduced levels of lipids, which normalize once the inflammation has resolved, indicating that hypolipidemia is a consequence of inflammation [52]. The pathways by which inflammation decreases the levels of different lipids are uncertain. Inflammatory cytokines can induce phospholipase A2, which increases the production of LPC from phosphatidylcholine (Figure S1). Conversely, decreased lecithin-cholesterol acyl transferase activity in inflammation may lower levels of LPC and cholesteryl esters [1] (Figure S1). Low levels of LPC and phosphatidylcholine in patients with active IBD suggest that both lipid classes are dysregulated (Figure S1). However, the underlying mechanisms remain unclear.

LPC is commonly derived from phosphatidylcholine by cleavage with secretory phospholipase A2 [17], the serum activity of which is increased in IBD [53]. Low serum levels of LPC in patients with severe IBD suggest that this enzyme may not induce LPC production. The association between IBD severity and levels of free fatty acids, which are produced in this reaction, is unclear [54,55] (Figure S1). This suggests that LPC processing may be enhanced. Autotaxin converts LPC to lysophosphatidic acid [56] (Figure S1), and its expression was increased in the inflamed colon of IBD patients [57]. Autotaxin inhibition in mouse colitis improved disease severity, but this beneficial effect does not appear to be related to reduced lysophosphatidic acid production [8]. To our knowledge, the levels of lysophosphatidic acid and autotaxin in the blood of IBD patients have not been investigated, and further studies are needed to show whether this is relevant to low LPC levels.

Drugs known to induce remission in IBD, such as glucocorticoids [27], inhibit secretory phospholipase A2 [28]. The patients treated with corticosteroids did indeed have lower levels of LPC 20:3 and 22:5. Similar levels of CRP and calprotectin were observed in patients with and without corticosteroid therapy, and there were no differences in the additionally analyzed lipid levels between these cohorts. The distribution of sex, age, BMI, and the number of patients with CD and UC treated with this drug was similar. The cause of lower levels of LPC 22:5 during corticosteroid therapy is currently unknown. Blocking TNF also impairs the activity of phospholipase A2 [30], but this did not affect the levels of LPCs. This suggests that this pathway does not contribute to the lower levels of LPC species observed during corticosteroid therapy. Glucocorticoid use in patients with rheumatoid arthritis caused higher serum LPC levels in women [58], but our cohort was too small for sex-specific analyses. Further study is needed to determine whether the associations between glucocorticoid treatment and serum LPC levels are related to sex and the underlying disease.

Our study has several limitations. Albumin levels were not available for analysis, and the overall sample size was modest. Although sex-specific analyses were not performed, LPC species levels did not differ between males and females, suggesting no major sex-related differences in our cohort. Similarly, LPC profiles did not differ between CD and UC; however, larger studies are warranted to confirm these findings, particularly since LPC species mostly correlated with CRP in patients with UC. Finally, as all the patients were recruited from a single center within the same geographic region, the generalizability of our findings requires confirmation in larger, multi-center cohorts.

In conclusion, our study demonstrates that levels of most LPC species, as well as phosphatidylcholine, are reduced in patients with severe IBD and elevated fecal calprotectin, reflecting an association with hypolipidemia in active disease. These findings demonstrate the interplay between lipid metabolism and intestinal inflammation in IBD. The overlap in levels of LPC between patients with mild IBD and healthy controls means that LPC species cannot be used as a marker for diagnosing IBD.

## Figures and Tables

**Table 1:** Statistical tests used and data presentation in the figures and tables.

Statistical tests	Mann–Whitney U-test (two-group comparisons) Kruskal–Wallis test (multiple group comparisons) Spearman correlation (associations between variables)
Graphs	Boxplots (median, minimum, and maximum values)
	Boxplots highlight outliers with circles and asterisks
Normality testing	Shapiro–Wilk tests (*p* < 0.05 for all but LPC 16:1, 18:1, 18:2, 20:4, 22:4, and 22:5) and non-parametric tests were used for all analyses [44]
Correction for multiple comparisons	*p*-values were adjusted by multiplying by 13 (total number of LPC species analyzed)
Significance threshold	*p* < 0.05
Data in Tables	Median, minimum, and maximum values
Software used	IBM SPSS Statistics 26.0 (IBM Corp., Armonk, NY, USA, released 2019)

**Table 2:** Description of the patients with inflammatory bowel disease (IBD). The median, minimum, and maximum values are shown.

Characteristics	IBD	Normal Values
Number (females/males)	57 (26/31)	
Age (years)	41.2 (19.1–69.9)	
Body mass index (kg/m^2^)	24.1 (15.5–44.3)	
C-reactive protein (mg/L)	2 (0–144)	<5
Fecal calprotectin (µg/g)	58 (0–1616)	<80
Aspartate aminotransferase (U/L)	25 (10–35)	Females: 0–35 Males: 0–50
Alanine aminotransferase (U/L)	18 (7–63)	Females: 0–35 Males: 0–50
Gamma-glutamyl transferase (U/L)	23 (8–100)	Females: <40 Males: <60
Alkaline phosphatase (U/L)	65 (38–142)	Females: 35–104 Males: 40–129
Bilirubin (mg/dL)	0.4 (0.1–1.9)	Females: 0–0.9 Males: 0–1.4

**Table 4:** Spearman’s correlation coefficients for the correlation of LPC species with cholesterol, triglycerides, and phosphatidylcholine in patients with IBD. * *p* < 0.05, ** *p* < 0.01, and *** *p* < 0.001.

LPC	Cholesterol	Triglycerides	Phosphatidylcholine
15:0	0.525 ***	0.427 *	0.542 ***
16:0	0.687 ***	0.570 ***	0.612 ***
16:1	0.459 **	0.450 **	0.526 ***
18:0	0.754 ***	0.488 **	0.659 ***
18:1	0.437 **	0.236	0.430 *
18:2	0.345	0.108	0.385 *
18:3	0.388 *	0.198	0.476 **
20:3	0.454 **	0.501 ***	0.449 **
20:4	0.342	0.318	0.243
20:5	0.318	0.234	0.335
22:4	0.300	0.119	0.200
22:5	0.348	0.402 *	0.315
22:6	0.477 **	0.226	0.411 *
Total LPC Level	0.601 ***	0.402 *	0.549 ***
CRP	−0.421 *	−0.116	−0.437 *
Calprotectin	−0.468 **	−0.259	−0.484 **

**Table 3:** Median, minimum, and maximum LPC concentration (in nmol/mL) of controls and patients with IBD. There were no significant differences between these groups.

LPC	Control			IBD		
	Median	Minimum	Maximum	Median	Minimum	Maximum
15:0	1.74	1.06	3.97	2.13	0.65	5.76
16:0	144.60	102.63	248.97	195.99	74.13	542.58
16:1	4.36	2.59	11.22	5.22	1.40	10.22
18:0	52.91	19.37	105.06	76.61	20.09	201.21
18:1	42.85	19.41	61.57	55.11	19.54	101.51
18:2	58.98	28.41	78.54	60.65	14.85	154.88
18:3	1.33	0.67	3.46	1.32	0.29	3.05
20:3	4.68	3.10	8.99	6.09	1.31	15.93
20:4	11.50	6.36	20.23	15.64	4.02	33.53
20:5	1.19	0.47	5.92	1.54	0.33	4.90
22:4	0.78	0.31	1.38	1.01	0.32	1.63
22:5	1.04	0.34	1.70	1.26	0.28	2.76
22:6	2.37	1.39	5.93	3.30	1.13	10.35
Total LPC	344.32	193.78	500.44	430.45	145.25	986.22

## Data Availability

Data is contained within the article or Supplementary Materials.

## Supplementary Materials

The following supporting information can be downloaded at https://www.mdpi.com/article/10.3390/jcm14155485/s1. Figure S1: Serum phosphatidylcholine, lysophosphatidylcholine, and cholesteryl ester levels in patients with active IBD are reduced; Figure S2: Serum LPC levels of patients with increasing fecal calprotectin as a marker of disease activity; Table S1: Description of the patients with Crohn’s disease and patients with ulcerative colitis; Table S2: Spearman’s correlation coefficients for the correlation between LPC species and the age and body mass index of patients with IBD; Table S3: Median, minimum, and maximum LPC concentration (in nmol/mL) of patients with Crohn’s disease and patients with ulcerative colitis. Table S4: Spearman’s correlation coefficients for the correlation of LPC species with CRP and fecal calprotectin in patients with IBD; Table S5: Spearman’s correlation coefficients for the correlation of LPC species with CRP and fecal calprotectin in patients with Crohn’s disease and patients with ulcerative colitis, Table S6: Spearman’s correlation coefficients for the correlation between LPC species and cholesterol, triglycerides, and phosphatidylcholine in patients with Crohn’s disease and patients with ulcerative colitis.

## Abbreviations

The following abbreviations are used in this manuscript:

BMI	Body mass index
CD	Crohn’s disease
CRP	C-reactive protein
IBD	Inflammatory bowel disease
LPC	Lysophosphatidylcholine
PC	Phosphatidylcholine
UC	Ulcerative colitis

## References

[1] Feingold K.R., Grunfeld C., Feingold K.R., Anawalt B., Blackman M.R., Boyce A., Chrousos G., Corpas E., de Herder W.W., Dhatariya K., Dungan K., Hofland J. (2000). The Effect of Inflammation and Infection on Lipids and Lipoproteins. Endotext.

[2] Wiesner P., Leidl K., Boettcher A., Schmitz G., Liebisch G. (2009). Lipid profiling of FPLC-separated lipoprotein fractions by electrospray ionization tandem mass spectrometry. J. Lipid Res..

[3] Pakiet A., Kobiela J., Stepnowski P., Sledzinski T., Mika A. (2019). Changes in lipids composition and metabolism in colorectal cancer: A review. Lipids Health Dis..

[4] Lee E.G., Yoon Y.C., Yoon J., Lee S.J., Oh Y.K., Kwon S.W. (2021). Systematic Review of Recent Lipidomics Approaches Toward Inflammatory Bowel Disease. Biomol. Ther..

[5] Bauset C., Gisbert-Ferrandiz L., Cosin-Roger J. (2021). Metabolomics as a Promising Resource Identifying Potential Biomarkers for Inflammatory Bowel Disease. J. Clin. Med..

[6] Rogler G., Singh A., Kavanaugh A., Rubin D.T. (2021). Extraintestinal Manifestations of Inflammatory Bowel Disease: Current Concepts, Treatment, and Implications for Disease Management. Gastroenterology.

[7] Agouridis A.P., Elisaf M., Milionis H.J. (2011). An overview of lipid abnormalities in patients with inflammatory bowel disease. Ann. Gastroenterol..

[8] Alhouayek M., Ameraoui H., Muccioli G.G. (2021). Bioactive lipids in inflammatory bowel diseases—From pathophysiological alterations to therapeutic opportunities. Biochim. Biophys. Acta Mol. Cell Biol. Lipids.

[9] Chen H., Li W., Hu J., Xu F., Lu Y., Zhu L., Shen H. (2023). Association of serum lipids with inflammatory bowel disease: A systematic review and meta-analysis. Front. Med..

[10] Sappati Biyyani R.S., Putka B.S., Mullen K.D. (2010). Dyslipidemia and lipoprotein profiles in patients with inflammatory bowel disease. J. Clin. Lipidol..

[11] Yan D., Ye S., He Y., Wang S., Xiao Y., Xiang X., Deng M., Luo W., Chen X., Wang X. (2023). Fatty acids and lipid mediators in inflammatory bowel disease: From mechanism to treatment. Front. Immunol..

[12] de Souza H.S., Fiocchi C. (2016). Immunopathogenesis of IBD: Current state of the art. Nat. Rev. Gastroenterol. Hepatol..

[13] Gajendran M., Loganathan P., Catinella A.P., Hashash J.G. (2018). A comprehensive review and update on Crohn’s disease. Dis. Mon..

[14] Zhao M., Feng R., Ben-Horin S., Zhuang X., Tian Z., Li X., Ma R., Mao R., Qiu Y., Chen M. (2022). Systematic review with meta-analysis: Environmental and dietary differences of inflammatory bowel disease in Eastern and Western populations. Aliment. Pharmacol. Ther..

[15] Tshikudi D.M., Bernstein C.N., Mishra S., Ghia J.E., Armstrong H.K. (2025). Influence of biological sex in inflammatory bowel diseases. Nat. Rev. Gastroenterol. Hepatol..

[16] Chang M.C., Lee J.J., Chen Y.J., Lin S.I., Lin L.D., Jein-Wen Liou E., Huang W.L., Chan C.P., Huang C.C., Jeng J.H. (2017). Lysophosphatidylcholine induces cytotoxicity/apoptosis and IL-8 production of human endothelial cells: Related mechanisms. Oncotarget.

[17] Liu P., Zhu W., Chen C., Yan B., Zhu L., Chen X., Peng C. (2020). The mechanisms of lysophosphatidylcholine in the development of diseases. Life Sci..

[18] Nakano T., Raines E.W., Abraham J.A., Klagsbrun M., Ross R. (1994). Lysophosphatidylcholine upregulates the level of heparin-binding epidermal growth factor-like growth factor mRNA in human monocytes. Proc. Natl. Acad. Sci. USA.

[19] Drobnik W., Liebisch G., Audebert F.X., Frohlich D., Gluck T., Vogel P., Rothe G., Schmitz G. (2003). Plasma ceramide and lysophosphatidylcholine inversely correlate with mortality in sepsis patients. J Lipid Res..

[20] Murgia A., Hinz C., Liggi S., Denes J., Hall Z., West J., Santoru M.L., Piras C., Manis C., Usai P. (2018). Italian cohort of patients affected by inflammatory bowel disease is characterised by variation in glycerophospholipid, free fatty acids and amino acid levels. Metabolomics.

[21] Guan S., Jia B., Chao K., Zhu X., Tang J., Li M., Wu L., Xing L., Liu K., Zhang L. (2020). UPLC-QTOF-MS-Based Plasma Lipidomic Profiling Reveals Biomarkers for Inflammatory Bowel Disease Diagnosis. J. Proteome Res..

[22] Tefas C., Ciobanu L., Tantau M., Moraru C., Socaciu C. (2020). The potential of metabolic and lipid profiling in inflammatory bowel diseases: A pilot study. Bosn. J. Basic Med. Sci..

[23] Daniluk U., Daniluk J., Kucharski R., Kowalczyk T., Pietrowska K., Samczuk P., Filimoniuk A., Kretowski A., Lebensztejn D., Ciborowski M. (2019). Untargeted Metabolomics and Inflammatory Markers Profiling in Children With Crohn’s Disease and Ulcerative Colitis-A Preliminary Study. Inflamm. Bowel Dis..

[24] Jonas A. (2000). Lecithin cholesterol acyltransferase. Biochim. Biophys. Acta.

[25] Woodruff T.M., Arumugam T.V., Shiels I.A., Newman M.L., Ross P.A., Reid R.C., Fairlie D.P., Taylor S.M. (2005). A potent and selective inhibitor of group IIa secretory phospholipase A2 protects rats from TNBS-induced colitis. Int. Immunopharmacol..

[26] Pruzanski W., Stefanski E., Vadas P., Ramamurthy N.S. (1997). Inhibition of extracellular release of proinflammatory secretory phospholipase A2 (sPLA2) by sulfasalazine: A novel mechanism of anti-inflammatory activity. Biochem. Pharmacol..

[27] Barrett K., Saxena S., Pollok R. (2018). Using corticosteroids appropriately in inflammatory bowel disease: A guide for primary care. Br. J. Gen. Pract..

[28] Nakano T., Ohara O., Teraoka H., Arita H. (1990). Glucocorticoids suppress group II phospholipase A2 production by blocking mRNA synthesis and post-transcriptional expression. J. Biol. Chem..

[29] Allgayer H., Stenson W.F. (1988). A comparison of effects of sulfasalazine and its metabolites on the metabolism of endogenous vs. exogenous arachidonic acid. Immunopharmacology.

[30] van Dullemen H.M., Wolbink G.J., Wever P.C., van der Poll T., Hack C.E., Tytgat G.N., van Deventer S.J. (1998). Reduction of circulating secretory phospholipase A2 levels by anti-tumor necrosis factor chimeric monoclonal antibody in patients with severe Crohn’s disease. Relation between tumor necrosis factor and secretory phospholipase A2 in healthy humans and in active Crohn’s disease. Scand. J. Gastroenterol..

[31] Hrabovsky V., Zadak Z., Blaha V., Hyspler R., Karlik T., Martinek A., Mendlova A. (2009). Cholesterol metabolism in active Crohn’s disease. Wien. Klin. Wochenschr..

[32] Vermeire S., Van Assche G., Rutgeerts P. (2006). Laboratory markers in IBD: Useful, magic, or unnecessary toys?. Gut.

[33] Romanato G., Scarpa M., Angriman I., Faggian D., Ruffolo C., Marin R., Zambon S., Basato S., Zanoni S., Filosa T. (2009). Plasma lipids and inflammation in active inflammatory bowel diseases. Aliment. Pharmacol. Ther..

[34] Sakurai T., Saruta M. (2023). Positioning and Usefulness of Biomarkers in Inflammatory Bowel Disease. Digestion.

[35] Siddiqui I., Majid H., Abid S. (2017). Update on clinical and research application of fecal biomarkers for gastrointestinal diseases. World J. Gastrointest. Pharmacol. Ther..

[36] Sands B.E. (2015). Biomarkers of Inflammation in Inflammatory Bowel Disease. Gastroenterology.

[37] Sturm A., Maaser C., Calabrese E., Annese V., Fiorino G., Kucharzik T., Vavricka S.R., Verstockt B., van Rheenen P., Tolan D. (2019). ECCO-ESGAR Guideline for Diagnostic Assessment in IBD Part 2: IBD scores and general principles and technical aspects. J. Crohns Colitis.

[38] Liebisch G., Schmitz G. (2009). Quantification of lysophosphatidylcholine species by high-throughput electrospray ionization tandem mass spectrometry (ESI-MS/MS). Methods Mol. Biol..

[39] Bligh E.G., Dyer W.J. (1959). A rapid method of total lipid extraction and purification. Can. J. Biochem. Physiol..

[40] Liebisch G., Lieser B., Rathenberg J., Drobnik W., Schmitz G. (2004). High-throughput quantification of phosphatidylcholine and sphingomyelin by electrospray ionization tandem mass spectrometry coupled with isotope correction algorithm. Biochim. Biophys. Acta.

[41] Horing M., Ejsing C.S., Hermansson M., Liebisch G. (2019). Quantification of Cholesterol and Cholesteryl Ester by Direct Flow Injection High-Resolution Fourier Transform Mass Spectrometry Utilizing Species-Specific Response Factors. Anal. Chem..

[42] Weigand K., Peschel G., Grimm J., Horing M., Krautbauer S., Liebisch G., Muller M., Buechler C. (2024). Serum Phosphatidylcholine Species 32:0 as a Biomarker for Liver Cirrhosis Pre- and Post-Hepatitis C Virus Clearance. Int. J. Mol. Sci..

[43] Horing M., Ejsing C.S., Krautbauer S., Ertl V.M., Burkhardt R., Liebisch G. (2021). Accurate quantification of lipid species affected by isobaric overlap in Fourier-Transform mass spectrometry. J. Lipid Res..

[44] Nahm F.S. (2016). Nonparametric statistical tests for the continuous data: The basic concept and the practical use. Korean J. Anesthesiol..

[45] Chen S., Yin P., Zhao X., Xing W., Hu C., Zhou L., Xu G. (2013). Serum lipid profiling of patients with chronic hepatitis B, cirrhosis, and hepatocellular carcinoma by ultra fast LC/IT-TOF MS. Electrophoresis.

[46] Ripolles Piquer B., Nazih H., Bourreille A., Segain J.P., Huvelin J.M., Galmiche J.P., Bard J.M. (2006). Altered lipid, apolipoprotein, and lipoprotein profiles in inflammatory bowel disease: Consequences on the cholesterol efflux capacity of serum using Fu5AH cell system. Metabolism.

[47] Law S.H., Chan M.L., Marathe G.K., Parveen F., Chen C.H., Ke L.Y. (2019). An Updated Review of Lysophosphatidylcholine Metabolism in Human Diseases. Int. J. Mol. Sci..

[48] Bazarganipour S., Hausmann J., Oertel S., El-Hindi K., Brachtendorf S., Blumenstein I., Kubesch A., Sprinzl K., Birod K., Hahnefeld L. (2019). The Lipid Status in Patients with Ulcerative Colitis: Sphingolipids are Disease-Dependent Regulated. J. Clin. Med..

[49] Kontush A., Lhomme M., Chapman M.J. (2013). Unraveling the complexities of the HDL lipidome. J. Lipid Res..

[50] Cameron K., Nguyen A.L., Gibson D.J., Ward M.G., Sparrow M.P., Gibson P.R. (2025). Review Article: Albumin and Its Role in Inflammatory Bowel Disease: The Old, the New, and the Future. J. Gastroenterol. Hepatol..

[51] Salguero S., Rojo D., Berenguer J., Gonzalez-Garcia J., Fernandez-Rodriguez A., Brochado-Kith O., Diez C., Hontanon V., Virseda-Berdices A., Martinez J. (2020). Plasma metabolomic fingerprint of advanced cirrhosis stages among HIV/HCV-coinfected and HCV-monoinfected patients. Liver Int..

[52] Sleutjes J.A.M., Roeters van Lennep J.E., Boersma E., Menchen L.A., Laudes M., Farkas K., Molnar T., Kennedy N.A., Pierik M.J., van der Woude C.J. (2021). Systematic review with meta-analysis: Effect of inflammatory bowel disease therapy on lipid levels. Aliment. Pharmacol. Ther..

[53] Minami T., Tojo H., Shinomura Y., Tarui S., Okamoto M. (1992). Raised serum activity of phospholipase A2 immunochemically related to group II enzyme in inflammatory bowel disease: Its correlation with disease activity of Crohn’s disease and ulcerative colitis. Gut.

[54] Jiang J.J., Chen L., Sun R., Yu T., Jiang S.Y., Chen H. (2023). Characterization of serum polyunsaturated fatty acid profile in patients with inflammatory bowel disease. Ther. Adv. Chronic Dis..

[55] Piotrowska M., Binienda A., Fichna J. (2021). The role of fatty acids in Crohn’s disease pathophysiology—An overview. Mol. Cell. Endocrinol..

[56] Tokumura A., Majima E., Kariya Y., Tominaga K., Kogure K., Yasuda K., Fukuzawa K. (2002). Identification of human plasma lysophospholipase D, a lysophosphatidic acid-producing enzyme, as autotaxin, a multifunctional phosphodiesterase. J. Biol. Chem..

[57] Hozumi H., Hokari R., Kurihara C., Narimatsu K., Sato H., Sato S., Ueda T., Higashiyama M., Okada Y., Watanabe C. (2013). Involvement of autotaxin/lysophospholipase D expression in intestinal vessels in aggravation of intestinal damage through lymphocyte migration. Lab. Investig..

[58] Fu J., Cuppen B.V., Welsing P.M., van Wietmarschen H., Harms A.C., Berger R., Koval S., Fritsch-Stork R.D., Bijlsma J.W., Hankemeier T. (2016). Differences between serum polar lipid profiles of male and female rheumatoid arthritis patients in response to glucocorticoid treatment. Inflammopharmacology.

