# Supplementary material for: Decline in Serum Lysophosphatidylcholine Species in Patients with Severe Inflammatory Bowel Disease
Source: J Clin Med. 2025 Aug 4;14(15):5485. doi: 10.3390/jcm14155485 (PMC12347743; doi:10.3390/jcm14155485)
Supplement: Supplementary file 1 [file jcm-14-05485-s001.zip › jcm-3743984-supplementary.pdf]

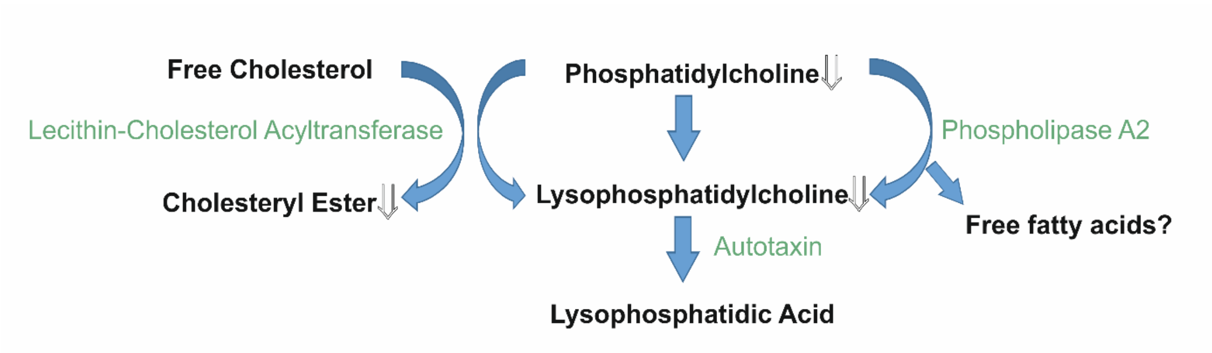

Figure S1. Serum phosphatidylcholine, lysophosphatidylcholine and cholesteryl ester levels in patients with active IBD are reduced. The enzymes catalysing the conversions shown are given in green. It is uncertain whether levels of free fatty acids are changed in IBD..

Table S1. Description of the patients with Crohn´s disease and patients with ulcerative colitis. The median, minimum, and maximum values are shown. There were no significant differences between the cohorts.

Characteristics	Crohn´s Disease	Ulcerative Colitis
Number (females/males)	40 (21/19)	17 (5/12)
Age (years)	41.4 (19.1 – 69.9)	35.5 (20.0 – 66.6)
Body mass index (kg/m²)	24.1 (17.5 – 44.3)	24.1 (15.5 – 40.4)
C-reactive protein (mg/L)	4 (0 – 44)	2 (0 – 144)
Fecal calprotectin (µg/g)	66 (0 – 1527)	54 (19 – 1616)
Aspartate aminotransferase (U/L)	24 (10 – 35)	25 (12 – 35)
Alanine aminotransferase (U/L)	19 (10 – 62)	16 (7 – 63)
Gamma-glutamyl transferase (U/L)	23 (10 – 74)	24 (8 – 100)
Alkaline phosphatase (U/L)	67 (43 – 121)	61 (38 – 142)
Bilirubin (mg/dL)	0.5 (0.2 – 1.9)	0.4 (0.1 – 1.1)

Table S2. Spearman's correlation coefficients for the correlation between LPC species and the age and body mass index of patients with IBD. ** $p < 0.01$.

LPC	Age	Body Mass Index
15:0	0.279	-0.092
16:0	0.364	-0.011
16:1	0.258	-0.056
18:0	0.452**	0.074
18:1	0.296	-0.134
18:2	0.198	-0.167
18:3	0.234	-0.087
20:3	0.337	0.093
20:4	0.226	0.001
20:5	0.206	-0.064
22:4	0.100	0.105
22:5	0.278	-0.005
22:6	0.135	-0.066
Total LPC	0.364	-0.081

Table S3. Median, minimum and maximum LPC concentration (in nmol/mL) of patients with Crohn's disease and patients with ulcerative colitis. There were no significant differences between these groups.

LPC	Crohn's disease			Ulcerative colitis		
	Median	Minimum	Maximum	Median	Minimum	Maximum
15:0	2.30	1.03	5.76	1.86	0.65	3.99
16:0	196.69	98.40	542.58	186.66	74.13	277.87
16:1	5.60	2.42	10.22	4.87	1.40	9.30
18:0	77.24	37.54	201.21	70.79	20.09	99.96
18:1	56.20	24.43	101.51	47.01	19.54	99.53
18:2	61.32	23.98	125.93	54.84	14.85	154.88
18:3	1.32	0.29	3.05	1.06	0.30	2.92
20:3	6.17	1.96	15.93	5.59	1.31	8.32
20:4	16.15	5.99	33.53	13.96	4.02	22.95
20:5	1.64	0.38	4.90	1.37	0.33	3.55
22:4	1.05	0.51	1.63	0.93	0.32	1.31
22:5	1.36	0.60	2.76	1.10	0.28	1.81
22:6	3.54	1.31	10.35	2.86	1.13	6.85
Total LPC	450.42	208.21	986.22	401.04	145.25	677.87

Table S4 Spearman's correlation coefficients for the correlation of LPC species with CRP and fecal calprotectin in patients with IBD. * p < 0.05, ** p < 0.01, *** p < 0.001.

LPC	CRP	Calprotectin
15:0	-0.356	-0.303
16:0	-0.432*	-0.372
16:1	-0.418*	-0.404*
18:0	-0.439*	-0.494*
18:1	-0.546***	-0.356
18:2	-0.595***	-0.360
18:3	-0.547***	-0.516**
20:3	-0.469**	-0.440**
20:4	-0.368	-0.244
20:5	-0.427*	-0.383*
22:4	-0.229	-0.160
22:5	-0.351	-0.259
22:6	-0.379	-0.181
Total LPC	-0.535***	-0.418*

Table S5 Spearman's correlation coefficients for the correlation of LPC species with CRP and fecal calprotectin in patients with Crohn´s disease and patients with ulcerative colitis. * p < 0.05, ** p < 0.01, *** p < 0.001.

LPC	CRP	Calprotectin	CRP	Calprotectin
	Crohn´s Disease		Ulcerative Colitis	
15:0	-0.189	-0.231	-0.706*	-0.424
16:0	-0.275	-0.291	-0.768**	-0.586
16:1	-0.326	-0.340	-0.620	-0.549
18:0	-0.195	-0.394	-0.838***	-0.662*
18:1	-0.490*	-0.313	-0.690*	-0.466
18:2	-0.508*	-0.295	-0.731*	-0.449
18:3	-0.439	-0.455*	-0.749**	-0.581
20:3	-0.372	-0.374	-0.725*	-0.654
20:4	-0.257	-0.153	-0.679*	-0.507
20:5	-0.265	-0.386	-0.741**	-0.402
22:4	-0.175	-0.039	-0.406	-0.453
22:5	-0.306	-0.231	-0.598	-0.380
22:6	-0.253	-0.079	-0.706*	-0.404
Total LPC	-0.386	-0.354	-0.800**	-0.542

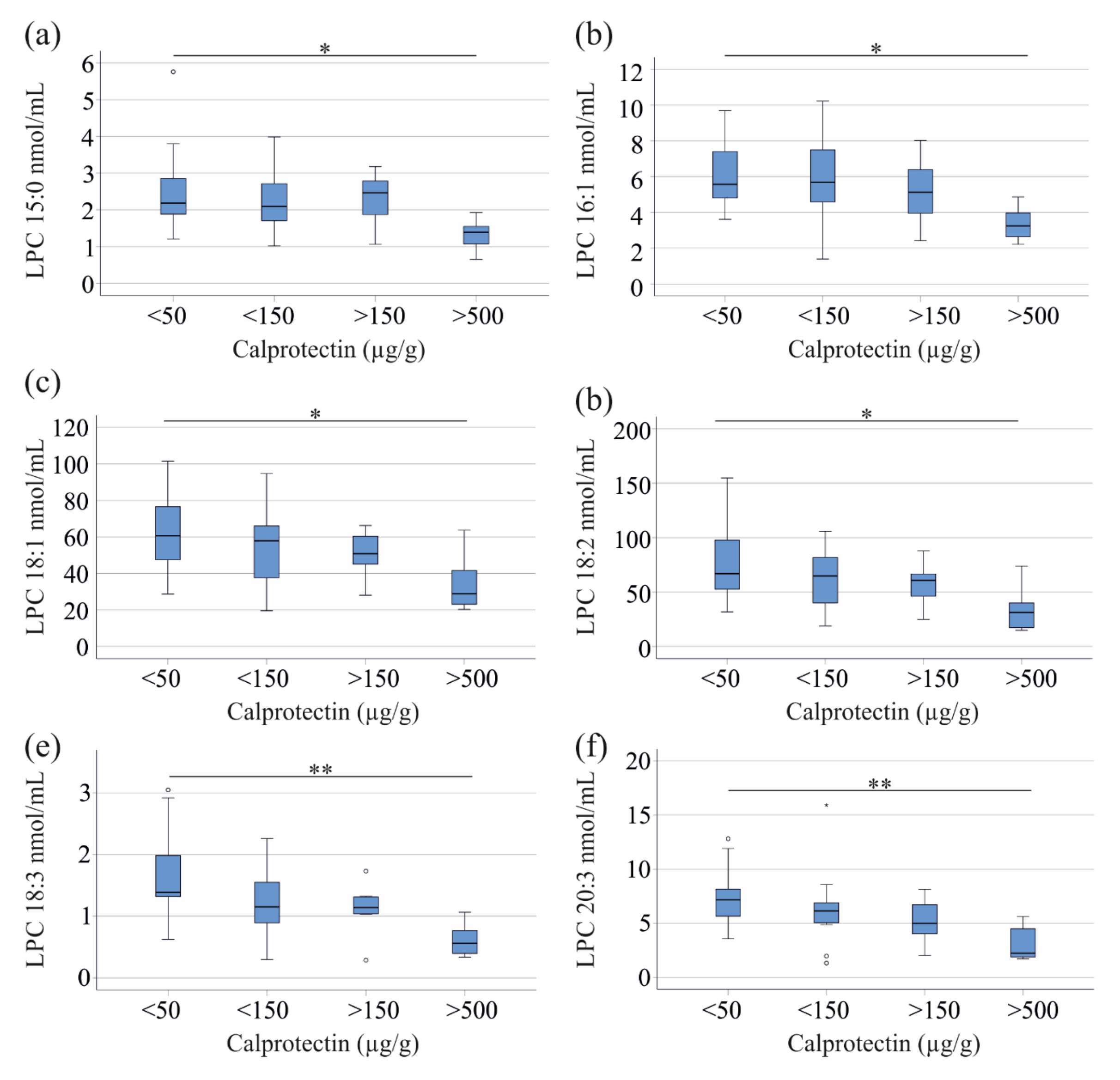

Figure S2. Serum LPC levels of patients with increasing fecal calprotectin as a marker of disease activity. (**a**) LPC 15:0; (**b**) LPC 16:1; (**c**) LPC 18:1; (**d**) LPC 18:2; (**e**) LPC 18:3, and (**f**) LPC 20:3 of IBD patients stratified for fecal calprotectin levels. * $p < 0.05$, ** $p < 0.01$.

Table S6. Spearman's correlation coefficients for the correlation between LPC species and cholesterol, triglycerides and phosphatidylcholine in patients with Crohn´s disease and patients with ulcerative colitis * p < 0.05, ** p < 0.01, *** p < 0.001.

LPC	Cholesterol	Triglycerides	Phosphatidylcholine	Cholesterol	Triglycerides	Phosphatidylcholine
	Crohn´s Disease			Ulcerative Colitis		
15:0	0.420	0.427	0.410	0.711*	0.520	0.740**
16:0	0.562**	0.572**	0.434	0.897***	0.603	0.880***
16:1	0.315	0.474***	0.317	0.662*	0.314	0.846***
18:0	0.679	0.483*	0.536***	0.853***	0.451	0.875***
18:1	0.289	0.172	0.258	0.650	0.365	0.767**
18:2	0.112	-0.045	0.180	0.708*	0.444	0.738**
18:3	0.165	0.138	0.252	0.721*	0.314	0.826***
20:3	0.276	0.469	0.255	0.792**	0.598	0.789**
20:4	0.156	0.245	0.068	0.672*	0.466	0.610
20:5	0.129	0.246	0.116	0.586	0.196	0.679*
22:4	0.192	0.050	0.113	0.542	0.201	0.385
22:5	0.211	0.335	0.168	0.600	0.576	0.600
22:6	0.341	0.090	0.284	0.654	0.434	0.586
Total LPC	0.459	0.369	0.367	0.806***	0.500	0.848***
CRP	-0.207	-0.027	-0.303	-0.749**	0.370	-0.782**
Calprotectin	-0.325	-0.129	-0.318	-0.799**	-0.549	-0.770**
